# Earable RCC: Development of an Earphone-Type Reliable Chewing-Count Measurement Device

**DOI:** 10.1155/2018/6161525

**Published:** 2018-02-12

**Authors:** Kazuhiro Taniguchi, Hisashi Kondo, Toshiya Tanaka, Atsushi Nishikawa

**Affiliations:** ^1^Graduate School of Information Sciences, Hiroshima City University, 3-4-1 Ozukahigashi, Asaminami-ku, Hiroshima, Hiroshima 731-3194, Japan; ^2^Wearable Media Laboratory, eRCC Co. Ltd., 21-3 Motomachi, Naka-ku, Hiroshima, Hiroshima 730-8504, Japan; ^3^SAGA-KEN Medical Centre KOSEIKAN, 400 Nakabaru, Kasemachi, Saga, Saga 840-8571, Japan; ^4^Faculty of Textile Science and Technology, Shinshu University, 3-15-1 Tokida, Ueda, Nagano 386-8567, Japan

## Abstract

Gastric cancer patients having undergone gastrectomy are at a high risk of becoming malnourished owing to decreased gastric function. To prevent malnutrition, patients need to thoroughly chew a mouthful of food at least 30 times. For these gastrectomy patients requiring dietary support, we developed a chewing-count measurement device named earable RCC using an earphone-type sensor. Experiments to evaluate the performance of this device were conducted on six healthy volunteers who participated in “gum-chewing tests” and “almond-eating tests.” The precision calculated based on the results was ≥0.958, indicating that the earphone-type chewing-count measurement device could experimentally distinguish chewing from other actions. In addition, the recall calculated from the test results was ≥0.937, showing that the device does not miss chewing actions and can accurately count the number of chews with high probability at the timing of chewing. The experimental results also imply that earphone-type sensors may be used to measure swallowing, occlusal force, and tongue motion. Our future plans include clinical testing of the earphone-type chewing-count measurement device to determine its utility in patients who have undergone gastrectomy. We also intend to expand the application of this device for use in other patients to aid in dementia prevention and dietary support.

## 1. Introduction

Gastric cancer patients who have undergone gastrectomy surgery are at a high risk of becoming malnourished because of decreased gastric function [1]. To prevent malnutrition, improvements in the method of meal intake are important. One such improvement is to slowly and thoroughly chew a mouthful of food about 30 times before ingesting it. Verbal instructions accompanied by materials such as pamphlets on chewing carefully are given to the patients; however, chewing depends subjectively on the patient and conducting the actual practice of slowly and thoroughly chewing before ingestion is often difficult. If a patient cannot slowly and thoroughly chew before swallowing, problems such as food quickly flowing into the intestines, inability of the body to adapt, and early dumping syndrome arise. By being able to visualize the practice of chewing thoroughly and applying it for patient support, the quality of life of patients having undergone gastrectomy (postgastrectomy patients) may be improved. We believe that visualizing the practice of chewing thoroughly can enhance the eating habits of patients and are trying to implement this at clinical sites. Meeting the “chewing thoroughly” metric can be made possible by quantifying the number of chews.

To improve the meal intake method and determine the number of chews, data obtained and recorded, using a chewing-count measurement device is preferable over that obtained and recorded by the patients themselves. This is because gathering and recording of data by the patients place undue burden on them, and the data may not be objective. Current devices to measure the number of chews include a camera that records mouth movements [2]; tooth-embedded sensors [3], EMG [4], and piezoelectric strain gauge sensors [5] attached to the skin surface; microphones that detect chewing noises [6–9]; and accelerometers that recognize the movement of skin caused by chewing [10, 11]. However, these devices were not developed specifically for dietary support of postgastrectomy patients, and each tool has drawbacks when applied to such patients. For instance, capturing mouth movements by a camera to measure chewing activity impinges on a patient's privacy. Tooth-embedded sensors to capture the number of chews are invasive and not well accepted by patients. EMG and piezoelectric strain gauge sensors that measure the number of chews dependent on muscle and skin movements require the sensors to be placed on the patient's skin, increasing the discomfort of patients and impeding their ability to eat meals. In addition, microphones that detect chewing noises tend to be ineffective due to ambient background noise. Accelerometers can detect whether meal intake is occurring or not but are unsuitable for accurately measuring the number of chews. Moreover, the design of all of these devices does not permit chewing measurement results to be attainable in real time to patients and physicians.

Therefore, we have been conducting research and development with respect to a chewing-count measurement device that uses an earphone-type sensor to provide dietary support for postgastrectomy patients [12–14]. In this paper, we describe the mechanics of the device and present the results from performance evaluation experiments. In our previous research, we focused on the distinctive pattern of variation associated with consuming meals (chewing), mainly with the aim of healthcare monitoring. We developed an earphone-type sensor (wearable device) and demonstrated its effectiveness in obtaining robust measurements during meals [15].

## 2. Materials and Methods

### 2.1. Earphone-Type Chewing-Count Measurement Device

In this research, we aimed to develop a chewing-count measurement device that met the following five criteria:

- Providing a measurement accuracy (precision and recall) of ≥90%
- Not impeding the patient's eating activities: less burden of wearing the device, number of chews measured without placing the sensor or device in the mouth (measurements can be taken even if there is food in the mouth), movements of the muscles and joints used for chewing (e.g., cheek joints and temporalis muscle) not obstructed by the device, and small and lightweight devices
- Protecting patient privacy
- Being easy to operate and facilitating handling without specialized knowledge
- Revealing the number of chews in real time to patients and physicians, recording meal contents as images, and presenting past meal contents (images) and measurements in graphical format concurrently

**Figure 1:**
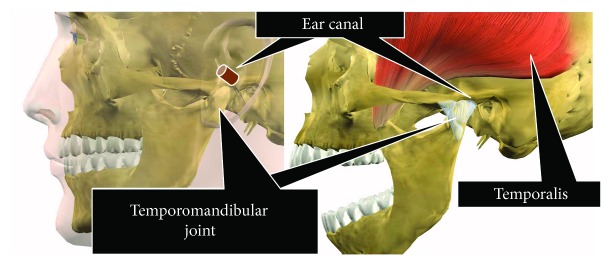
Relationship between chewing and changes in ear canal shape.

Chewing occurs via the movement of the temporalis muscle and temporomandibular joint. Based on the anatomical positional relationship between the temporalis muscle and temporomandibular joint shown in Figure 1, chewing activity changes the shape of the ear canal near the temporalis muscle and temporomandibular joint. We measured the change in shape of the ear canal due to chewing with an earphone-type sensor, and using the obtained results, we developed a device that determines the number of chews. The earphone-type sensor contains an optical distance sensor that uses light for taking measurements. Using this method, chewing count can be obtained without irritating sensitive ears.

**Figure 2:**
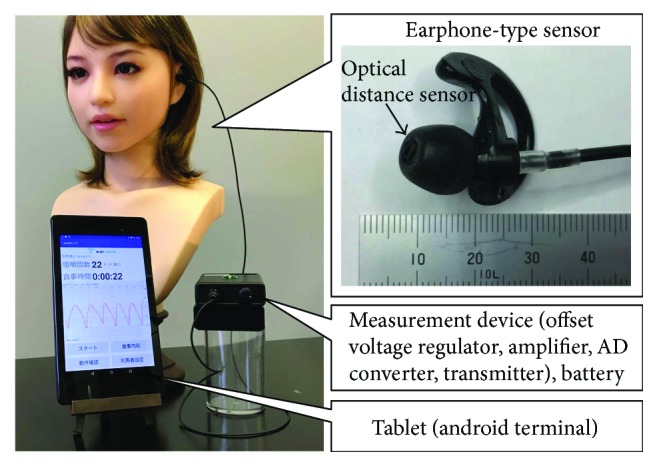
Outer appearance of earphone-type chewing-count measurement device (earable RCC).

**Figure 3:**
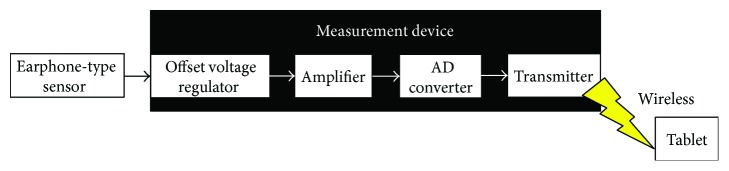
Configuration of the chewing-count measurement device.

**Figure 4:**
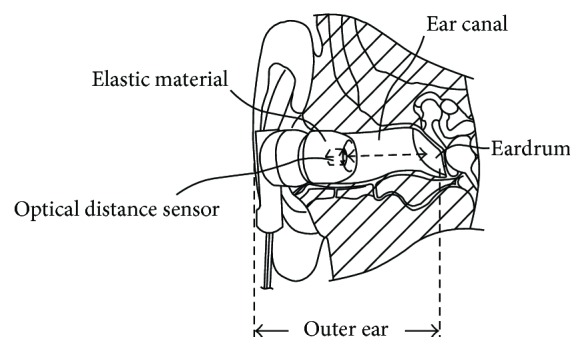
Measurement principle for changes in the shape of the ear canal.

**Figure 5:**
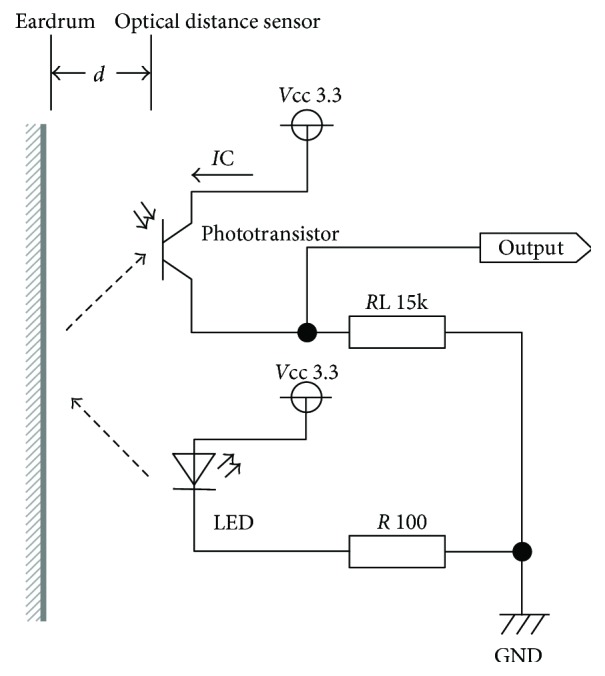
Electronic circuit around the optical distance sensor.

The outer appearance of the earphone-type chewing-count measurement device named earable RCC is shown in Figure 2. The configuration of the device is shown in Figure 3. In this device, the earphone-type sensor is attached to either the right or left ear, and the movement of the ear canal is measured. The earphone-type sensor has the same shape as an inner-ear-type earphone and is fitted with an optical distance sensor, QRE1113 (Fairchild Semiconductor International Inc., California, USA), which has an infrared LED and a phototransistor built in. Infrared light emitted from LED is transmitted within the ear canal, and the reflected light is captured by the phototransistor. From the obtained data, movements of the ear canal during chewing can be measured (Figure 4). The electronic circuitry around the optical distance sensor is shown in Figure 5. In this diagram, when distance *d* between the eardrum and optical distance sensor is short, the amount of light reflected from the eardrum is large and the output voltage increases. Similarly, when distance *d* is long, the amount of reflected light is small and the output voltage decreases.

The earphone-type sensor to be inserted into the ear canal was made in two sizes, small and medium, based on the sensors that are commercially available. The sensor was also constructed for both the right and the left ears for each size.

The earphone-type sensor was connected to a measurement instrument with a cable. The instrument was 110 × 75 × 25 mm in size and weighed 115 g; it was small enough to be unobtrusive on a dining table. A voltage of 3.3 V DC was supplied from the instrument to the earphone-type sensor, and the output data from the sensor were detected by the offset voltage regulator of the instrument. The amount of offset voltage measured by the sensor was adjusted to a central value after analog to digital (AD) conversion of the signal received by the offset voltage regulator of the instrument. The adjustment was based on the following formula: central value of AD convertible range = 3.3 V (power supply voltage of the AD converter) ÷ 2 = 1.65 V. The offset voltage adjustment is required to correct for differences in this parameter caused by dissimilarities in ear canal shape among individuals. The value (waveform) measured by the sensor is an amplitude based on the offset voltage. Only the amplitudes were magnified in the signal after offset voltage adjustment because the offset voltage was kept fixed by the amplifier. The amplification level could be magnified up to 40 times by a knob (variable resistor) on the measurement instrument. The analog signal after amplification was converted to a digital signal by the AD converter with a sampling frequency of 250 Hz and a 10-bit resolution. The converted digital signal was sent by a transmitter (Bluetooth 2.1) to a tablet (ASUS Nexus 7, Bluetooth 3.0). By the aid of an application software that we built, the tablet displayed and recorded the waveforms of values sent from the transmitter, number of chews, and duration of measurement. The software can be installed on an android terminal (e.g., tablets and smartphones) supporting version 5.0 and above. When chewing is detected by this software, the information is displayed on the tablet with concurrent sounds emitted through the speaker to the user. In this software, one chew was deemed to have occurred when the peak-to-peak waveform value exceeded 0.4 V (1.65 ± 0.2 V). We chose this threshold of 0.4 V based on trial and error. Before measuring the number of chews using the earphone-type chewing-count measurement device, the amplification level was adjusted using the knob on the instrument such that the peak-to-peak waveform on the tablet was 0.4 V or more. This adjustment was made while the subjects moved their mouths when empty as if chewing and when eating foods used for testing.

The software that we developed manages patient information via ID numbers, thereby protecting the privacy of patients. Using a camera embedded in the android terminal, images of the meal contents can be taken and recorded. This information can be linked to the measurements of chewing counts. The chewing-count data can be searched by patient ID, and the measurements can also be displayed in a graphical format. The software and hardware are designed for intuitive operation, and the device can easily be handled without specialized knowledge. These functions of the chewing-count measurement device are useful for the dietary support of patients and were implemented according to requests from clinicians.

We conducted experiments to evaluate the measurement accuracy (precision and recall) of the chewing-count measurement device involving subjects described in Section 2.2 for evaluation experiments outlined in Section 2.3.

### 2.2. Subjects

Six volunteers (men and women between 21 and 43 years of age; mean age, 28.2 years) served as subjects and were individually identified as A to F. All subjects were healthy, had no history of surgery such as gastrectomy, and had never received dietary counselling. Subjects were those who could chew without problems, did not have pain in their teeth or jaws, and did not have subjective symptoms of fatigue; volunteers who were undergoing orthodontic or medical treatment were excluded. Furthermore, the ear sensor had to fit on the subjects without it being too large or small; subjects who did not have symptoms of ear pain or fatigue were selected, whereas those under treatment were excluded.

This study was approved by the Shinshu University Ethical Committee on Human Science Research. An adequate explanation of the study was given to the subjects in advance, and consent for research participation was obtained.

### 2.3. Evaluation Experiments

Subjects participated in experiments involving chewing two types of food. Prior to the experiment, the subjects selected either a small or medium earphone-type sensor that fit on their left or right ear.

For the gum-chewing experiment, subjects were requested to refrain from other actions such as taking food into their mouth or swallowing and to focus on chewing the gum only. According to a report by the Chewing Gum Association of Japan, the number of chews for one 3 g stick of gum is approximately 550. Gum sold in Japan is often in the form of 1.5 g sticks; thus, we assumed that the number of chews for this type of gum would be half that for a 3 g stick or approximately 275. Xylitol Oratect Gum (Lotte Co. Ltd., Tokyo, Japan) was used in this experiment. Prior to measuring the number of chews, the subjects, while using the earphone-type chewing-count measurement device, placed a 1.5 g stick of gum in their mouth and started chewing. At that time, the researcher adjusted the amplification level by turning the knob on the measuring instrument such that the peak-to-peak waveform displayed on the tablet was ≥0.4 V. Once this adjustment had been made, the subjects expelled and disposed of the gum. For the experiment, one fresh 1.5 g stick of gum was taken in the mouth and chewed 300 times; during this period, the number of chews was measured by the earphone-type chewing-count measurement device. Concurrently, the subjects reported the number of chews they made with a manual counter. The researcher also used a manual counter to note the number of times that the chewing-count measurement device indicated chewing, although the subject was not chewing.

For the eating experiment, the subjects ate 10 almonds one by one. We chose to use almonds because one almond can be eaten in a mouthful, and because the size and hardness of almonds do not vary much compared with those of other foods, they are readily available as size-selected products. Almonds are crushed by chewing, and their hardness changes greatly by chewing. Therefore, in this experiment, it was possible to include actions such as taking food in the mouth, chewing the food hard until soft, and swallowing food. Unsalted roasted almonds obtained from a convenience store (7-Eleven Japan Co. Ltd., Tokyo, Japan) were used. A preliminary experiment conducted with subject A indicated that the number of almonds that could be eaten by chewing approximately 300 times (the same number as for gum chewing) was 10; therefore, 10 almonds were used to minimize differences in the number of chews between the gum-chewing and almond-eating experiments. Furthermore, dietary support instructions to postgastrectomy patients indicated that they should chew a mouthful of food about 30 times. Thus, when subjects chew one almond 30 times, the total number of chews for 10 almonds eaten one by one is 300 times. Prior to measuring the number of chews, the subjects, while using the earphone-type chewing-count measurement device, chewed one almond. At that time, the researcher adjusted the amplification level by turning the knob on the measuring instrument such that the peak-to-peak waveform displayed on the tablet was ≥0.4 V. For the experiment, 10 almonds were eaten one by one until fully consumed; during this period, the number of chews was measured by the earphone-type chewing-count measurement device. Concurrently, the subjects reported the number of chews they made with a manual counter. The researcher also used a manual counter to note the number of times that the chewing-count measurement device indicated chewing, although the subject was not chewing.

After all the experiments, a survey was administered by interviewing the subjects. All of the experiments were performed with the subjects sitting in a chair, with a table placed in front of them. On this table, one dish containing two sticks of gum, a second dish with 11 almonds, the measuring instrument, one android tablet, and one manual counter were placed.

Prior to the experiment, the researcher informed the subjects about ingredients in the gum to be used and explained that the gum contained gelatin, which can be an allergen. In addition, the researcher notified the subjects that the almonds to be used had been processed in a manufacturing facility where products, including eggs, milk, wheat, peanuts, and shrimp, were handled. Moreover, after notification, the researcher confirmed with the subjects that they did not have allergic reactions to the gum or almonds used.

During all experiments, the subjects were requested not to drink fluids such as water. If fluids were ingested during the chewing measurement experiment, the results were invalidated, and the experiment was reperformed. The earphone-type sensor was cleaned and disinfected with ethanol before and after use in the experiments.

## 3. Results

We conducted the gum-chewing and almond-eating experiments outlined above and obtained results on the number of chews determined by the chewing-count measurement device (recorded in the internal memory of the device) and the number of chews the subjects reported with a manual counter. In addition, the number of times that the chewing-count measurement device indicated chewing despite the subject not chewing was noted by the researcher using a manual counter. Results from a survey administered to the subjects in an interview format by the researcher were also acquired. Furthermore, the subject's chewing condition, waveform displayed on the measuring device, and the number of chews were visually observed, and the results were recorded by the researcher.

Table 1 shows the results from each subject when chewing one 1.5 g stick of gum 300 times. Precision *p* values in Table 1 were calculated using (1) and indicated the ratio of “the number of times chewing was indicated by the device when the subject was chewing” to “the number of times chewing was indicated by the device.”

(1) $$\begin{array}{c} p=\frac{TP}{TP+FP}. \end{array}$$

Here, “true positive (TP)” represents the number of times the device indicated chewing when the subject was actually chewing, and “false positive (FP)” represents the number of times the device indicated chewing despite the subject not chewing. In other words, TP is the number of times the device indicated chewing subtracted by the number of times the researcher recorded on the manual counter that the device indicated chewing despite the subject not chewing. FP is the number of times the researcher recorded on the manual counter that the device indicated chewing despite the subject not chewing.

Recall *r* values in Table 1 were calculated using (2) and indicated the ratio of “the number of times the device indicated chewing when the subject was actually chewing” to “the number of times the subject was actually chewing.”

(2) $$\begin{array}{c} r=\frac{TP}{TP+FN}. \end{array}$$

Here, “false negative (FN)” represents the number of times that the device failed to count the subject's actual chewing and is equal to the number of times the subject recorded on the manual counter that he/she was chewing subtracted by the number of times the chewing-count measurement device indicated chewing and added by the number of times the researcher recorded on the manual counter that the device indicated chewing despite the subject not chewing.

Table 2 shows a summary of the results from each subject when 10 almonds were eaten one by one. The same calculation methods as used for constructing Table 1 were used.

There was no need to reperform any of the chewing measurement experiments due to the ingestion of fluids by the subjects. In addition, all subjects selected an earphone-type sensor that fits in the right ear. Subjects A and B chose the small size, and subjects C to F chose the medium size.

## 4. Discussion

Based on Tables 1 and 2, precision was shown to be ≥0.958, indicating that the earphone-type chewing-count measurement device incorrectly identified chewing occurrences only a few times. In addition, based on Tables 1 and 2, recall was shown to be ≥0.937, indicating that after chewing was finished, the device could accurately count the number of chews with a high probability of not missing chews.

Based on Table 1, the chewing results from each subject were evaluated. First, the results for subject A indicate precision to be 1.000; thus, when subject A was not chewing, the chewing-count measurement device correctly identified that chewing was not occurring. The precision in results obtained from subjects B to E was the same as that for subject A. The precision in results acquired from subject F was 0.987. At this instance, the device incorrectly recognized chewing when subject F swallowed saliva while chewing gum. The cause of erroneously identifying swallowing as chewing appears to be that the measured change in shape of the ear canal (peak-to-peak waveform in the earphone-type sensor) during swallowing was larger in subject F than that in the other participants. The value quantified for swallowing in the other subjects was less than or equal to the threshold value set using the measurement instrument, whereas the value exceeded the threshold value in subject F, resulting in the misidentification of chewing. These results from subject F were obtained by the earphone-type sensor, suggesting that the technology can be used to measure swallowing in addition to chewing. Chewing is often conducted in succession, but swallowing is intermittent. Based on this, we believe that chewing could be distinguished from swallowing by amplifying the measurement waveform of the sensor and conducting frequency analysis of the waveform. Thus, in the future, we plan to clarify and quantify the relationship between swallowing and changes in the shape of the ear canal and to conduct research on swallowing measurements using the earphone-type sensor.

The chewing-count measurement device did not fail even once in detecting chewing in subjects A, C, E, and F, as evidenced by the finding of recall = 1.00 shown in Table 1. Recall values were 0.937 and 0.993 for subjects B and D, respectively. Compared with subject D, results from subject B indicated that a large number of chews (19 out of 300) were unrecognized by the device. These 19 undetected chews occurred consecutively in the latter half of the experiment. During this time, the waveform on the device was synchronized with chewing performed by subject B; however, the peak-to-peak value of the waveform fell below the threshold of 0.4 V; thus, the device did not recognize chewing. Based on the results of the postexperiment survey, it was discovered that subject B experienced fatigue near the end of chewing gum 300 times consecutively without rest and found it difficult to continue exerting occlusal force. Therefore, we need to take into consideration chewing fatigue when using the chewing-count measurement device for the dietary support of gastric cancer patients. The results also suggest that the device could be employed to measure occlusal force; thus, we have started research on this issue [16].

Based on Table 2, the eating results from each subject were evaluated. The results for subject B indicated precision to be 1.000; thus, when subject B was not chewing, the chewing-count measurement device correctly identified this. Precision values were 0.984, 0.958, 0.996, 0.990, and 0.966 for subjects A and C to F, respectively. Based on the results of the postexperiment survey, it was discovered that subject A had used his/her tongue to remove a piece of almond stuck to the back of a tooth, and the device had erroneously indicated this action as chewing. This result suggests that an earphone-type sensor is potentially applicable to the measurement of tongue movement. In addition, the results from subjects C–F revealed that swallowing was misidentified as chewing by the device.

The chewing-count measuring device did not fail even once in detecting chewing in subjects A, C, and F, as evidenced by the recall value being 1.00 in Table 2. Recall values were 0.974, 0.988, and 0.980 for subjects B, D, and E, respectively. In these instances, the device could not detect chewing on the brink of swallowing. This was probably due to the use of almonds as test food; because the almonds were crushed by chewing, the occlusal force needed for chewing decreased, and the values measured by the device on the brink of swallowing reached the threshold value or lower. Therefore, we plan to build on the above occlusal force measurement study not only toward the application of the device for occlusal force measurement but also toward device improvement to address the decreases in the accuracy of chewing measurements associated with changes in occlusal force due to jaw fatigue caused by chewing, eating, and grinding of food. In this experiment, the amplification level of the signal was set by turning the knob of the measurement instrument before the experiment. However, the signal amplitude was found to change depending on the extent of the subject's fatigue; thus, we need to reexamine how the amplification level is set when operating the device.

We developed an earphone-type chewing-count measurement device that is hands-free because the sensor is worn in the ear. Thus, the apparatus does not obstruct vision or require electrodes placed on the face, as in the past, and regular meal intake is possible. In addition, the device consists only of an earphone-type sensor, a small, lightweight measurement instrument (110 × 75 × 25 mm, 115 g weight), and a tablet terminal; therefore, it takes up little space and is not an obstacle when eating meals. The device displays in real time not only the number of chews but also the ear canal motion accompanying chewing as a waveform on the tablet terminal. The waveform associated with ear canal movements due to chewing can be considered a visual representation of the chewing action (jaw movements). In other words, subjects can “visualize” their chewing in real time while eating meals. The device can also record and retain in its memory the number of chews and waveforms of ear canal movements associated with chewing; thus, accumulation and analysis of the measurement results are subsequently possible. The device can emit sounds as each chew occurs. Because of the combination of visual display and sound, it is possible to recognize chewing action visually and aurally. This enables a subject to receive feedback on chewing movements aurally even if he/she is visually occupied with another action (e.g., common activities during mealtimes such as watching TV or reading the newspaper). We believe that by visualizing chewing movements and number of chews, postgastrectomy patients can direct their own chewing behavior in real time, resulting in improvement of dietary habits (an increase of the number of chews) and overcoming of postoperative complications.

Future plans include improving the accuracy of the earphone-type chewing-count device, developing the same earphone-type sensor for swallowing and occlusal force quantification [16], and conducting further research on measurements of tongue movement. To confirm the usefulness of the device, we plan to deploy it for postgastrectomy patients. In addition, because improvement in chewing is desirable for dementia prevention and diet support, the device will also be applied to these fields.

## 5. Conclusions

To aid the recovery of postgastrectomy patients, we developed a chewing-count measurement device that provides real-time visualization of chewing movements and the number of chews to the patient. This information is necessary for patients to self-monitor and improve their meal-intake behavior by increasing the number of chews before swallowing.

We developed a device that measures the number of chews using a wearable earphone-type sensor and displays the information on a tablet terminal in real time. The device was tested on 6 healthy volunteers who chewed gum or ate almonds; the results confirmed that the instrument can accurately count chewing without missing any chewing occurrences and can correctly distinguish behaviors other than chewing. The experimental results also suggest that the earphone-type sensor can be used to measure swallowing, occlusal force, and tongue movement in addition to chewing.

The chewing-count measurement device requires the subject to only wear an earphone-type sensor to measure chewing and thus does not obstruct meal intake and allows “visualizing” and monitoring of the subject's chewing actions on a tablet screen in real time while eating.

Future plans include clinical application of the measurement device for postgastrectomy patients. In the future, we will expand this study to include a larger sample, particularly to include a wider age range of subjects. Furthermore, we would like to include at-home use as a potential context of this device and conduct experiments in real-life scenarios such as talking or drinking while chewing to examine how these actions affect the measurement accuracy and make modifications to the device as necessary. Further, broader application of the device, such as for dementia prevention and dietary support, is being considered. The measurement range for the earphone-type sensor will also be expanded for the quantification of swallowing, occlusal force, and tongue movement in addition to chewing.

## Figures and Tables

**Table 1:** Results from chewing experiment. Results from chewing one 1.5 g stick of gum 300 times. For precision, the numerator is TP and the denominator is the sum of TP and FP. For recall, the numerator is TP and the denominator is the sum of TP and FN.

Subject	Precision	Recall
A	1.000 = 300/300	1.000 = 300/300
B	1.000 = 281/281	0.937 = 281/300
C	1.000 = 300/300	1.000 = 300/300
D	1.000 = 298/298	0.993 = 298/300
E	1.000 = 300/300	1.000 = 300/300
F	0.987 = 300/304	1.000 = 300/300

**Table 2:** Results from eating experiment. Results from eating 10 almonds one by one. For precision, the numerator is TP and the denominator is the sum of TP and FP. For recall, the numerator is TP and the denominator is the sum of TP and FN.

Subject	Precision	Recall
A	0.984 = 300/305	1.000 = 300/300
B	1.000 = 331/331	0.974 = 331/340
C	0.958 = 207/216	1.000 = 207/207
D	0.996 = 255/256	0.988 = 255/258
E	0.990 = 291/294	0.980 = 291/297
F	0.966 = 230/238	1.000 = 230/230
